# 

**Published:** 1883-01-20

**Authors:** 


Abortion and premature labor' prevention of by asafœtida.......... 429
Abscess, mammary, prevention of.......... 429
Acne.......... 16, 208
Aconite and belladonna in Erysipelas.......... 147
Agoraphobia.......... 14
Air-passages, foreign body in.......... 229
Albuminuria in phthisis.......... 30
do. chloral in.......... 428
Alcoholism, coffee an antidote to.......... 106
Alebama, medical association of.......... 195
Alcohol, inhalations of in croupal diphtheria.......... 109
Aloes, externally.......... 273
Alimentation, nasal.......... 9
Alum for extinguishing fires.......... 181
American medical association.......... 241, 156
American surgical association.......... 276
American medical association, journal of the.......... 316
Amenorrhœa.......... 428
Amaurosis, quinine.......... 230
Anæsthesia of the pharynx.......... 57
Anæsthetics, new.......... 29
Anæsthesia, local.......... 473
Anæsthesia, local.......... 264
Anæsthetics, deaths from.......... 303
Angina pectoris, death.......... 28
Anatomy in Southern Medical College.......... 118
Anatomy, practical.......... 316
Anorexia.......... 154
Anti-spree mixture.......... 433
Anti-diphtheritic inhalations.......... 153
Aphthous sore mouth in infants.......... 233
Arbor-vitæ.......... 388
Association of american medical editors.......... 159
Asthma mixture.......... 74
Asthma, grindelia robusta in.......... 274
Aspidospermo Quebracho.......... 425
Apomorphia, in cases of poisoning,.......... 455
Application for removal of scars,.......... 474
Aunt Betsey Simpson's case.......... 69
Auto-transfusion in hemorrhage.......... 465
Avena Sativa in opium habit.......... 67

B

Bacteria of syphilis.......... 29
Bacteria, the latest about.......... 27
Baltimore medical association.......... 368
Bacillus of tubercle, Koch's.......... 198
Bacilli of tubercles only fat-crystals.......... 148
Balloon for Pictorial World.......... 191
Baldness, remedy for.......... 234
Belladonna in hernia.......... 189
Belladonna and aconite in erysipelas.......... 147
Bee's sting, fatal effects from.......... 257
Benzoate of iodine.......... 465
Berberis aquifolium in syphilis.......... 345
Bills, to collect.......... 110
Birth-rate, average.......... 111
Bladder, aspiration of.......... 21
Blood-letting.......... 380
Blindness, premature delivery for prevention of.......... 89
Boracic acid.......... 26
Borax for whitening garments.......... 235
Boracic acid cotton.......... 35
Bovine virus disapproved.......... 224
Book notices....39, 78, 119, 159. 199, 238, 280, 358, 439, 479
Boroglyceride in treatment of diseases of eye and ear.......... 259
Brom. of ethyl as an anæsthetic for short and painful operations.......... 58
Brom. of ethyl, method of administering.......... 61
Bronchitis, expectorant mixtures in.......... 235
Breast, in the puerperal.......... 132
Bronchocele, extirpation of, and results.......... 425
Bronchitis, acute.......... 194
Bryonia.......... 435
Burns, bi carb soda for.......... 187
Burroughs, Chas. J., M. D., diphtheria.......... 401

C

Case of doubtful diagnosis.......... 346
Cannabis indica in epistaxis.......... 147
Cannabis indica in menorrhagia.......... 349, 390
Cannabis indica in hemorrhagia.......... 383
Cannabine, tannate of.......... 274
Cancer, hypophosphite of lime in.......... 353
Capsicum enemata in opium poisoning.......... 98
Capsicum, powdered, in rheumatism.......... 189
Calomel in diphtheria.......... 149, 389
Calomel in croup.......... 307
Catheters, broken in urethra.......... 95
Cathartics for infants.......... 273
Cathartic pill, a new compound.......... 155
Carbolic acid in piles and other diseases.......... 201
Carbolic acid in piles and fistula.......... 267
Cardiac neurasthenia.......... 192
Catarrh, nasal.......... 309
Calculus, renal.......... 285
Cancer, ergot in.......... 22
Candles from kerosene.......... 232
Calomel, influence of on fermentation, etc.......... 422
Cancer remedy.......... 427
Carbolic acid, poisoning by.......... 181
Catarrh, tseatment of.......... 433
Cephalhæmatoma in the new born.......... 340
Cephalalgia.......... 83
Cellulose as a dressing.......... 386
Celerina.......... 70
Cart, the Frazier road, (of interest to doctors).......... 475
Cascara amarga.......... 466
Character and climate.......... 472
Chills, a valuable mixture for.......... 73
Chloroform with air, action of.......... 387
Chloral, new use of.......... 313
Cholera.......... 148, 156, 279, 356
Cholera—What is it?.......... 289
Cholera morbus.......... 343
Cholera infantum.......... 390
Cholera, sporadic, treatment of.......... 385
Cholera infantum, resorcine in.......... 396
Cholera, Squibb,s remedy for.......... 394
Chamomile in infantile diarrhœa.......... 147
Chest development by exercise.......... 150
Chest, aspiration of, difficulties in.......... 22
Cholagogue formula.......... 155
Chills, chronic.......... 474
Chromic acid.......... 188
Chloroform, death from.......... 215
Chloroform—What shall we do with it?.......... 369
Child-bed, laxative in.......... 314
Chloroform, hypodermically, in toothache.......... 270
Chloral hydrate, therapeutics of.......... 108
Chloroform, an adjuvant to.......... 428
Chloral in Albuminuria.......... 428
Christianity and Science.......... 152
Cinchonidine, benzoate of.......... 424
Climate and character.......... 472
Code, at american medical association.......... 276, 279
Colon, irrigation of.......... 7
College of medical practitioners, St. Louis.......... 37
Contagious diseases, isolatiou of.......... 269
Convulsions, puerperal.......... 25, 63
Convulsions, infantile.......... 107
Colic in children.......... 179
Colic, quinine for.......... 230
Conjunctivitis, purulent, new treatment for.......... 106
Cologne antiseptic.......... 113
Cough mixture, palatable.......... 275
Cough medicines.......... 113
Coffee, physiological effect of.......... 382
Coffee, iodo-ferrated syrup of.......... 430
Coffee, syrup of.......... 394
Coffee as an antidote to alcoholism.......... 106
Coca, viburnum and celery, combination of.......... 253

Codein, phosphate of.......... 447
Code, new.......... 469
Cosmetic preparations.......... 154
Constipation, remedy for.......... 193
Corrosive sublimate.......... 226
Confidential communications.......... 227
Corn silk, pharmaceutical preparations of.......... 294
Consumption, diagnosis of.......... 230
Consumption, antiseptic breathing for.......... 275
Cordial, hop.......... 234, 314
Cordial, blackberry.......... 313
Colchicum in gout.......... 307
Commotio retinæ, from blows to the eye.......... 386
Convulsions, puerperal, treatment of.......... 468
Colors, variety of.......... 352
Creasote wine.......... 394
Creasote wine.......... 473
Croup and diphtheria.......... 463
Croup, vegetable nature of.......... 350
Croup, treatment of.......... 268
Cystitis, chrouic, Reliquet's injectioa for.......... 146

D

DaCosta, on salts of nickel.......... 427
Deaths of physicians.......... 399
Death from swallowing silver half dollar.......... 256
Delirium tremens, treatmeut of.......... 227
Decision in relation to surgeon's fee.......... 141
Dispensatory, U S.......... 77, 118
Diphtheria, bi-chloride of mercury in.......... 457
Diphtheria and croup.......... 463
Diphtheria.......... 264, 401
Diphtheria, croupal, alcohol inhalations in.......... 109
Diphtheria, treatment of.......... 121
Diphtheria, calomel in.......... 149, 389
Diphtheria, turpentine in.......... 270
Diarrhœa.......... 194
Diarrhœa, oxide of zinc in.......... 433
Diarrhœa pills.......... 114
Diarrhœa, infantile, chamomile in.......... 147
Diarrhœa, mineral acid in.......... 187
Diarrhœa, percussion of colon in.......... 262
Diarrhœa, chronic, oxlde of zinc in.......... 315
Dislocations of the thigh, new methods of reducing, 143
Diabetes, cure of.......... 150
Diabetes, the teeth in.......... 263
Disinfectants.......... 347
Doctor, good.bye to.......... 99
Doctor, a smart.......... 270
Doctor, rare chance for a.......... 475
Doctor's diploma before a jury.......... 373
Doctors, country and city.......... 377
Doctors, number of in world.......... 29
Doctors, how to treat.......... 198
Don't.......... 308
Doses, small.......... 30
Dreams, shortness of time in.......... 391
Drug case, beautiful.......... 157
Dyspepsia.......... 41, 182
Dyspepsia, flatulent.......... 314, 396
Dysentery.......... 192, 193
Dysentery, a new treatment of.......... 185
Dysentery, bilious.......... 193
Dysmenorrhœa mixture.......... 315
Dysmenorrhœa, gelremium in.......... 113

E

Earth, gradual cooling of.......... 311
Earth, tremors of the.......... 312
Earache, remedy for.......... 395
Ectrotic, petroleum as in small-pox.......... 470
Eczema of the genitalia, pruritus and leucorrhœa, 142
Eczema aurium.......... 19
Eczema, chronic.......... 204
Eczema.......... 274
Eczema,.......... 459
Eczema of heart in children.......... 474
Eczema rubrum of face and scalp.......... 435
Eczema of leg, hands, nipple, beard, eyelids.......... 205
Eczema of hands.......... 355
Eczema of face.......... 393
Eczema, to allay itching in.......... 395
Eclampsia.......... 216
Electricity, signalling by.......... 72
Electricity for paralysis and other diseases.......... 103
Elyctricity in parlor, kitchen and everywhere.......... 72
Electricity, a non-conductor of.......... 190
Electricity, various uses of.......... 191
Electricity, stopping steam engines by.......... 191
Electric light, portable.......... 195
Electrical lighting, progress of.......... 71
Electrical exhibition at Paris, curious accident at... 376
Electric light.......... 152
Electric light in surgery.......... 183
Emmenagogues.......... 34, 234
Embalming, new methods of.......... 110
Epistaxis, value of cannabis indica in.......... 147
Erysipelas, facial, abortive treatment of.......... 29
Erysipelas, aconite and belladonna in.......... 147
Erythema simplex.......... 17
Erysipelas, local treatment of.......... 454
Ergot in congestive headache.......... 349
Eserine solutions, permanency of.......... 390
Ethics, New York code of.......... 77
Ethyl, brom. of as an anæsthetic in short and painful operations.......... 58
Ethyl, brom. of, method of administering.......... 61
Eucalyptus globulus.......... 228
Experiment, a curious.......... 112
Eyelids, pain in.......... 83
Eye-water, Thompson's.......... 393

F

Faith cures.......... 359
Face, pain in one side of.......... 84
Facts worth knowing.......... 114
Faucial cough and vomiting.......... 85
Fever, malarial, treatment of.......... 33
Fever, hemorrhagic malarial.......... 65, 281
Fever, scarlet, sulphate of zinc a specific in.......... 96
Fever, typhoid.......... 182
Fever, therapeusis of remittent.......... 331
Fever, tephoid, salicylate of bismuth in.......... 337
Fever, marsh, the parasite of.......... 342
Fever, scarlet, application of vaseline in.......... 348
Fever, malarial, a new remedy for.......... 349
Fever, typhoid, pulse and temperature in.......... 389
Fever, typhoid, specific treatment of.......... 462
Fever, typhoid, treatment of.......... 464
Fever, typhoid, special indications for salicylate of soda in.......... 189
Fever, enteric, so-called specific treatment of.......... 167
Felon, how to cure.......... 217
Fætus, viability of the premature.......... 261
Fee, a dentists.......... 310
Fish bones, to remove from throat.......... 470
Fistula ani.......... 230
Flies, effect of castor oil plant on.......... 311
Flatulence.......... 283
Fox, Addison C., M.D.; Treatment of diphtheria.... 121

G

Gas poisoning.......... 471
Gaultheria, oil of.......... 325
Gallstones, olive oil in.......... 175
Gelsemium semper virens in tetanus.......... 148
Gelsemium in dysmenorrhœa.......... 113
Gelsemium, therapeutic action of.......... 871
Germination, limits of.......... 391
Georgia med. assoc'n.......... 76, 118, 164, 195, 357, 397
Glass, protective coating for.......... 472
Gelatine test for organisms in water.......... 431
Glottis, spasm of.......... 84
Glycerine.......... 268
Glycerine, internally.......... 462
Glycerine, internal effects of.......... 348
Gonorrhœa.......... 153
Gohorrhœa easily cured.......... 419
Gonorrhœa, hydrastis in.......... 426
Goitre, acute.......... 428
Gout, treatment of.......... 341
Gout, Sir Benj. Brodie's prescription for.......... 396
Grave-robbing.......... 37
Graveyard, a mastodon.......... 272
Greenley, T. B., M. D.: Kentncky med. soc'y.......... 161
do. American med. assoc'n.......... 241

Grindella robusta in asthma.... 274
Gums, lancing of.... 268

H

Hair-wash.... 74

Hair, effect of pilocarpin upon color of.... 390

Hæmaturia, malarial.... 339

Hæmoptysis.... 470

Harness polish.... 473

Hernia, reduction of.... 56

Hernia, belladonna in.... 189

Hernia, incarcerated, puzzling case of.... 22

Headache from kerosene lamps.... 72

Headache, congestive, ergot in.... 349

Headache, frontal, iodide of potassium in.... 142

Hemorrhage, auto-transfusion in.... 465

Hemorrhagic diathesis.... 260

Hemorrhagia, cannabis indica in.... 383

Hemorrhage, post partum, hot water in.... 218

Hecker, Prof. Carl Von, the great obstetrician.... 156

Heavy.... 158

Hospital for skin diseases, Phila.... 157

Hobbs, A. G., M.D.; Treatment of iritis.... 4

Hurricanes.... 32

Hydrocele, Dr. Cattaneo's treatment of.... 188

Hydrophobia, cure of.... 267

Hypophosphite of lime in cancer.... 353

Hydrastin in laryngeal phthisis.... 267

Hypnotics, paraldehyde and acetal as.... 384

Hypodermic use of stimulants.... 127

Hypertrophied spleen, iodoform in.... 473

I

Idiocy.... 20
Ileus, shot and insufflation in treatment of.... 268
Intra-uterine crying.... 36
In memoriam.... 198
Intestinal obstruction, successful laparotomy in.... 305
Inebriate asylnm.... 276
Infant feeding.... 406
Infants, aphthous sore mouth in.... 233
Insects, strength of.... 432
Inventions, burdensome.... 71
Inebriety, trance state in.... 122
Ink, a new invisible.... 275
Insanity, causes of.... 330
Insanity, prevention of.... 328
Iodine, benzoate of.... 465
Iodine, use of as stomachic sedative.... 208
Iodism.... 298
Iodism, treatment of.... 299
Iodoform in chronic pulmonary affections.... 35
Iodoform, the ooor of.... 233
Iodoform in hypertrophied spleen.... 473
Iodoform in spray form.... 263
Iodide of po.assium.... 296
Iodine painting in smallpox.... 388
Iron shot.... 190
Iron, showers of.... 351
Iron, hydrated oxide of.... 395
Iron rust spot remover.... 433
Iritis, treatment.... 4
Irritation, intestinal, from swallowing mouse.... 326
Itching, intense.... 396

J

Jaundice, jaborandi in.... 110
Johnson, Jno. Thad., M. D., remarkable case of fibro-cellular and cartilaginous tumor.... 448
Journalism, medical, as a teacher.... 227, 237
Jupiter, disappearance of spot on.... 312

K

Kairine, a substitute for quinine.... 310
Kalsomining fluid.... 275
Kerosene, candles of.... 232
Kentucky med. soc'y.... 161
Kidney, complete destruction of.... 286
Koch's reply to his critics.... 176
Kocher, notice of paper by.... 425

L

Laryngitis, acute.... 385
Larynx and trachea.... 171
Labor, case of difficult.... 49
Laparotomy for intestinal obstruction.... 305
Labor, anæsthetics in.... 334
Labor, ipecac in.... 344
Labor, to shorten term and lessen hours of.... 347
Laxative for gouty and rheumatic subjects.... 235
Lecture by Prof. Powell.... 475
Lecture to medical class at South'n Med. College, Oct., 1883.... 441
Lenses, some large.... 111
Listerism without spray.... 305
Lichen.... 207
Lightning rods.... 111
Light, an instantaneous.... 191
Living a double life.... 151
Liver, for torpid.... 353
Lochia, offensive, to correct.... 73
Low, J. H., M.D.; Uræmia.... 284
Logan, J. H., M.D.; Oil of Gaultheria.... 325
Lupus exedens, solar ray cautery in.... 86
Luxations, femoral, new point in diagnosis of.... 70
Lungs, tuberculosis of.... 233

M

Martinet, Dr., on typhoid fever in pregnancy.... 427
Malpractice, a bar to an action for.... 150
Machinist, an expert.... 312
Medical education of women in Russia.... 143
Medical practice, perils of.... 144
Med. soc'y of Tenn., transactions of....38, 318
Med. assoc'n of Missouri.... 78
Med. soc'y of Louisiana.... 119
Med. assoc'n of Georgia at Athens.... 156
Medical societies, the good of.... 158
Medical editors, American.... 276
Medical codes, etc.... 278
Medical ministry....317, 357
Med. soc'y, St. Louis, on the code.... 318
Med. assoc'n of Miss., transactions of.... 318
Med. soc'y of West Va., transactions of.... 319, 438
Medical colleges....356
Med. assoc'n of Texas, transactions of.... 438
Medicine, practical.... 302
Medicine, therapeutic action of small, frequently repeated doses of.... 321
Meteoric metallic particles.... 191
Meningitis, treatment of.... 217
Menstruation, painful.... 315
Menorrhagia, cannabis indica in....349, 390
Meniere's disease.... 103
Mercury, bi-chloride of, in diphtheria.... 457
Migraine, or hemicrania, remedy for.... 355
Milk, artificial.... 266
Milk, hot, as a restorative.... 469
Misers.... 31
Mineral waters.... 196
Moon and weather.... 32
Monkeys, syphilitic innoculation of.... 466
Moral training—true progress in medical schools.... 476
Mouse in child's stomach.... 326
Mullein in phthisis.... 266
Murder, ante-natal.... 343

N

Nasal alimentation.... 9
Narcotic, a new.... 110
Naphthaline as an antiseptic.... 28
Naphthalin in frostbites.... 426
Nausea, quinine.... 188
Nausea and vomiting in uterine affections.... 474
Naphthol for local sweating.... 354
Nævus, treatment of.... 424
Negro with tail.... 478
Neuralgia, intercostal electricity in.... 103
Neuralgia, treatment of obstinate.... 270
Neuralgia, cilliary.... 82
Neuralgia....372, 381
Neuralgia, muscæ volitantes and supra-orbital.... 81
Needle five years in the body.... 184
New York med. and surgical society.... 285
Nervine and anti-spasmodic.... 34
Negatives taken on the wing.... 126
Nickel, salts of.... 427
Nipple, to harden.... 75

Nipple, excoriated..... 354
Nipple, cracked..... 234
No, sir!..... 356
Nursing, new reasons for..... 387

O

Obstetric soc'y of Phila..... 361
Obstetrics, a remarkable case of..... 426
Octagonal religious services in the Eastern penitentiary, Phila..... 311
Olive oil in treatment of gallstones..... 175
Opium habit, successful treatment by avena sativa, 67
Opium poisoning, capsicum enemata in..... 98
Opium addiction, clinical notes on..... 221
Opium habit..... 246
Ophthalmic aphorisms..... 104
Os uteri, ulceration of..... 224
Ovaritis, chronic..... 139
Oxygen and nitrogen, liquid..... 431

P

Palestine, climate of..... 112
Parham, E. H. M., M.D.; Hemorrhagic malar'l fever, 281
Painkillers..... 354
Paper, luminous, how to make..... 72
Paper from moss..... 72
Paraldehyde and strychnine, antagonism of..... 463
Paraplegia caused by intestinal worms..... 429
Paints, how to mix..... 432
Parsley mixture, compound..... 394
Pertussis, treatment of..... 75
Petroleum, death from suffocation by..... 269
Petroleum..... 150
Petroleum, as an ectrotic in small-pox..... 470
Petroleum, in phthisis-consumption..... 467
Phthisis, laryngeal, hydrastin in..... 267
Phthisis, albuminuria in..... 30
Phthisis-consumption, petroleum in..... 467
Phthisis; mullein in..... 266
Phthisis: veratrum and arsenic in treatment of..... 146
Phthisis averted by typhoid fever..... 369
Pharynx, anæsthesia of..... 57
Photography and pockmarks..... 272
Photography of birds, etc., on wing..... 126
Physician, the..... 461
Physicians, taxing..... 37
Physicians, integrity in..... 437
Physician and the New Year..... 39
Pharmacy in America..... 399
Pharyngitis, follicular..... 434
Pharmacopia, U. S..... 436
Pills, Dr. Gross' neuralgic..... 33
Pills, diarrhœa..... 114
Pill, a new compound cathartic..... 153
Piles, bleeding..... 275
Piles, treatment of..... 137
Pickerel caught with tackle attached..... 432
Pigs, discovery of cause of disease in..... 189
Plaxiscindens, discovery of in oysters, etc..... 389
Placenta prævia, treatment of..... 183
Placenta, removal of..... 458
Pneumonia, a prognostic sign of..... 185
Pneumonia..... 178
Poisoning, apomorphia in..... 455
Poisoning, arnica..... 375
Poisoning, stramonium..... 309
Poison, blood..... 392
Poison, carbolic..... 108, 181
Poisoning, gas..... 471
Polish, for harness..... 473
Post-partum hemorrhage, hot water in..... 218
Potassium, iodide of in frontal headache..... 142
Potassium, chlorate of..... 178
Potassium, brom. of, for unpleasant effects of quinine..... 267
Powell, Prof., marriage of..... 77
Powell, T. S., M. D.:
Mineral waters, etc..... 196
In memoriam..... 198
Med. journalism as a teacher..... 237
Journalism as a teacher..... 277
Med. ministry..... 317, 357
Powell, Prof..... 356
Powell, T. S., M. D., true progress—moral training in medical schools..... 476
Powell, Prof., lecture by..... 475
Powell, Prof. T. S., lecture to medical class at Southern Med. College, Oct. 1883..... 441
Prehistoric remains discovered..... 232
Premium, a..... 316
Prescriptions in France, ownership of..... 429
Prehistoric men, discovery of..... 431
Premium of $100,000..... 478
Progress—true moral training in medical schools... 476
Prolapsus recti, new operation for..... 460
Prurigo..... 459
Pruritus ani..... 350
Pruritus vulvæ..... 74
Prussic acid, an insect that secretes..... 181
Psoriasis and eczema, salicylic acid in..... 52
Psoriasis..... 206
Psoriasis..... 460
Pulmonary affections, iodoform in..... 35
Puerperal convulsions, treatment of..... 468
Puerperal septicæmia, intra-uterine injections in treatment of..... 213

Q

Quassine, effects of..... 68
Quackery years ago..... 138
Quinine, hypodermic use of..... 34
Quinine, disguising taste of..... 74
Quinine, tasteless..... 75
Quinine in whooping-cough..... 186
Quinine nausea..... 188
Quinine, novel method of taking..... 194
Quinine amaurosis..... 230
Quinine used in colic..... 230
Quinine intoxication..... 269
Quinine, brom. of potassium for unpleasant effects of..... 267
Quinine, to hasten the action of..... 425
Quinine salts..... 410

R

Reader, to the..... 478
Rape during hypnotic slumber..... 141
Rats and mice, to kill..... 235
Reflex neurosis and nose diseases..... 81
Remedies, old and almost obsolete..... 450
Renewals..... 436
Resorcine in cholera infantum..... 396
Resorcine in treatment of purulent vaginitis..... 434
Restorative, hot milk as a..... 469
Reviews..... 479
Revivication..... 134
Rheumatism, acute..... 1, 93, 393
Rheumatism, use of electricity in chronic..... 103
Rheumatism, muscular, liniments in..... 315
Rheumatism aborted..... 347
Ring, how to remove..... 344
Roy, G. G., M.D.:
Acute rheumatism..... 1
Minute and frequently repeated doses of medicine..... 321
Unrecognized cause of gastro-intestinal irritation in child discovered..... 326
Rotheln..... 358

S

Salicylic acid cotton..... 35
Salicylic acid, elixir of..... 154
Salicylic acid in psoriasis and eczema..... 52
Salts, quinium..... 410
Scabies..... 207
School for practitioners..... 316
Scar, application for removal..... 474
Scarlatina, so-called equine..... 453
Scarlatina, greasing with fat bacon for..... 187
Science and Christianity..... 152
Sciatica, use of electricity in..... 103
Sensible..... 158
Secrets, professional..... 357
Septicæmia, puerperal, intra-uterine injections in.. 213
Shoulder, dislocation of..... 136

Shoes, the hygiene of........ 151
Signal service, benefit of........ 71
Singultus, specific for........ 235
Sight, good........ 389
Sims, Dr., death of........ 436
Skin affections, tartar emetic in........ 459
Skin diseases, per chloride of iron in........ 188
Skin diseases, treatment of commoner forms of........ 203
Smallpox, iodine painting in........ 388
Small-pox, petroleum as an ectrotic........ 470
Smallpox, to prevent pitting in........ 396
Southern Medical College Commencement........76, 115
Southern Medical College, Atlanta........236, 399
Southern World........ 236
Solar ray cautery, treatment of lupus exedens by... 86
Sodium, sulpho-carbolate in vomiting........ 140
Solanine in unripe or very old potatoes........ 136
Sodium and potassium, bromides of........ 258
Sore throat, clergymen's........ 274
Speculum discovered in ruins of Pompeii........ 143
Specific for sore throat........ 110
Spectroscope, the afflictions of........ 231
Spleen, hypertrophied, iodoform in........ 473
Sponge-grafting........ 142
Spermatorrhœa, treatment of........ 180
Squint, cure of without operation........ 257
S. S. S........ 395
Stephens, Gov. of Ga., death of........ 118
Stimulants, hypodermic use of........ 127
Styptic, a new vegetable........ 648
Steel, melting........ 190
Still-born, reviving the........ 338
Strychnine........ 379
Strychnine and paraldehyde, antagonism between.. 463
Stricture, urethral........ 388
Sthenic and asthenic conditions........ 249
Sun spot laws........ 272
Sulphate of zinc in scarlet fever........ 96
Surgeon's fee, recent decision as to........ 141
Submarine work........ 31
Surgical instruments, to protect........ 433
Swallowing a 5 franc piece........ 226
Sycosis........ 460
Syphilis vs. vaccine........ 426
System, the metric........ 113
Syphilis, a new remedy for........ 225
Syphilis, six years experience in treatment of........ 265
Syphilis, berberis aquifolium........ 345
Syphilis, general and special facts concerning........ 365
Syphilis from accidental causes........ 374
Syphilis, did it exist in America previous to discovery........ 350
Syphilitic innoculation of monkeys........ 466

T

Tape-worm, treatment for........ 11
Tænia solium, turpentine for........ 155
Tartar-emetic, in skin affections........ 459
Telagraph wires and spider webs........ 392
Telegraph, time by........ 190
Temperatures, comparative........ 114
Tetanus, gelsemium semporvirens in........ 148
Telephone, large........ 312
Telephone, inventor of........ 351
Telephone, first idea of........ 391
Teeth, extraction of deciduous........ 306
Teeth, effect of alum gargle upon........ 382
Tetter of the hands........ 396
Thompson's eye-water........ 393
Throat, sore, specific for........ 110
Throat, to remove fish bones from........ 470
Tinea tonsurans........ 207
Torticollis........ 286
Toe-nail, ingrowing........ 6
Tonsillitis and its treatment........385, 407
Tonsillitis, acute........ 389
Town, coldest in the world........ 311
Tonsils, enlarged........186. 390
Tonic, Baer's........ 270
Tornado, how to act in a........ 271
Toothache drops........ 114
Toothache, chloroform injections in........ 270
Trees, Australian........ 31
Tri-state med. assoc'n........ 276
Trepanning, cure of dementation by........ 232
Tracheotomy........ 105
Troches, Brown's........ 314
Trance in a hysterical woman........ 336
Tuberculossis, Koch's theory of........ 100
Tumor, remarkable case of fibro-celular and cartilaginus........ 448
Turpentine in diphtheria........ 270

U

U. S. Dispensatory........ 280
Underbidding, no more........ 475
Urticaria........ 460
Urticaria........18, 396
Urine, analyzing........156, 342
Uterine affections, medication in........ 192
Uterine affections, nausea and vomiting in........ 474

V

Vaccinia, from variola........ 30
Varicocele........ 130
Vaccination with saliva of calf........ 265
Vaccinating live stock........ 312
Vaginitis, new treatment for........ 73
Vanadium........ 31
Vanadium discovered in Arizona mine........ 152
Van Goidtsnoven, Dr. E.; Infant feeding........ 406
Varicocele, cure by excision of the venous plexus.. 420
Vertigo........ 108
Veratrum and arsenic in treatment of phthisis,........ 146
Vessel, a submarine........ 152
Venus, transit of........ 232
Venom, copperhead........ 351
Ventilation, household........ 352
Vomiting, sodium sulpho-carbolate in........ 140
Vomiting in pregnancy, pop-corn for........ 149
Volta prize........ 230

W

Water filter, cheap........ 272
Water, hot, treatment by Salisbury plan........ 412
Water, drinking, organic matter in........ 429
Warts, removal of........ 228
Warts, removal of by cauterization........ 268
Westmoreland, J. G., M.D.:
Treatment of piles........ 137
Carbolic acid in piles and other diseases........ 201
Westmoreland, Dr. Jno. G........ 156
Whooping-cough, bromides and chloral in........ 73
Whooping-cough, the bacillus of........ 145
Whooping-cough........ 149
Whooping-cough, quinine in........ 186
Whooping-cough specific........ 395
Whooping-cough, croton-chloral in the treatment of........430, 435
Wild chery, com. syrup of........ 313
Wine, creasote........ 473
Wood, metalized........ 471
Word, R. C., M.D.:
Ingrowing toe-nail........ 6
Taxing physicians........ 37
Clinical remarks on dyspepsia........ 41
Medical codes, etc........ 278
Women, health of criminal........ 99
Wounds, "dry-dressing" treatment of........ 415

Y

Young children, water in dietary of........ 417

Z

Zinc sulphate, a specific in scarlet fever........ 96

### EDITORIALS AND MISCELLANEOUS.

☞ See the Electric Brush advertisement—a novelty.

☞ See Battle & Co.'s new advertisement in this Journal; a staunch house and an enterprising one.

DRS. L. P. YANDELL AND MCMURTY are now in charge of the Louisville Medical News, Dr. Cottell having resigned.

DR. J. J. WOODWARD, ex-President of the American Medical Association, and one of tne consulting physicians in the case of ex-President Garfield, is reported dangerously ill.

SIR THOMAS WATSON —The death of Sir Thomas Watson is announced. He was an eminent physician and a fine writer. He reached the age of 90 years.

INTRA-UTERINE CRYING.—Dr. Harlow reports to the Michigan Medical News, a case in which the fœtus was heard to cry in the womb of the mother before delivery. We don't know so well about that.

PALMER'S PRACTICE OF MEDICINE.—The New York Medical Journal and Obstetric Review, gives a very unfavorable, and it seems to us, a somewhat over severe criticism upon Dr. Palmer's new work on practice.

THE NEW YORK POST-GRADUATE MEDICAL SCHOOL, it is said, has thus far met with gratifying success. The second term opened Jan. 8, 1882, and continues until April 28th, without intermission. It is believed that with its enlarged accommodations, improved facilities for instruction and increased corpse of teachers, it will meet with still greater success.

PARKE, DAVIS & CO.—We are pleased to acknowledge the New Year's greeting from Messrs. Parke, Davis & Co., the members of that splendid and enterprising Drug house at Detroit, Michigan. We cannot match the beautiful and elegant card upon which "The Happy New Year" is engraved, but we heartily return the same greeting and warmly reciprocate their kind and complimentary expressions for THE RECORD.

SCOTT & BOWNE.—We tender our warmest acknowledgements to Messrs. Scott & Bowne, of 108 and 110th Wootster street, New York, for the New Year's token sent us, a floral representation of an exquisitely beautiful Flower Basket. As staunch business men, and as manufacturing Chemists, they are highly esteemed by the profession. Their emulsion of Cod-Liver Oil with hypophosphites, and other preparations are excellent, and are coming into general use by the profession everywhere.

and contegrude scale. Both the mitric and common measures are used. We regret to notice that the doses are not given.

The work contains 488 octavo pages, and is sold in muslin at $4.00; in leather at $5.00

---

### *RECEIPTED.*

1881—Drs. J L Silman, R W Lovett.

1882—Drs. P P Terry, H T Shiell, C F Rogers, J W Hill, G A M Cook, J S Miller, E H Wright, J W Rickman, C W Bowling, P A Welhite, G R Dozier, C S Prustly. W W Culpepper, J A Gordon, J M Stansill, J R McQueen, J F Blanks, P S Anderson, A B Loving, W H Wilson, W B Maxwell, J H McCaleb, W E Walker, J W Talley, T L H Cook, H S Bruce, J M Lewis, R D Jackson, L T Boatright, A R Brewington, J H Green, A H Sellers, A Atkinson, J W Hoff, J Hysel. F Courtney, W B King, C H. Jones.

1883—Drs. J A Ardry, J H Wysong, J B Payne, T P Olliver, Lockwood Alison, Lib. Surg. Gen'l; A J Sewell, Nathan King, Geo W Clower, R L Hinton, John Geidine, R H Edwards, R E Toombs, J W Baker, S M Logan, E A Anderson, W J Oglesby, W T Beall, J T Cleveland.

